# Assessment of the Physicochemical, Antioxidant, Microbial, and Sensory Attributes of Yogurt-Style Products Enriched with Probiotic-Fermented *Aronia melanocarpa* Berry Juice

**DOI:** 10.3390/foods13010111

**Published:** 2023-12-28

**Authors:** Stavros Plessas, Ioanna Mantzourani, Antonia Terpou, Argyro Bekatorou

**Affiliations:** 1 Laboratory of Food Processing, Faculty of Agriculture Development, Democritus University of Thrace, 193 Pantazidou Str., 68200 Orestiada, Greece; imantzou@agro.duth.gr; 2 Department of Agricultural Development, Agri-Food, and Natural Resources Management, School of Agricultural Development, Nutrition & Sustainability, National and Kapodistrian University of Athens, Evripos Campus, 34400 Evia, Greece; 3 Department of Chemistry, University of Patras, 26504 Patras, Greece; abekatorou@upatras.gr

**Keywords:** yogurt, probiotics, *Aronia melanocarpa*, bioactive, supplement, functional food

## Abstract

The aim of this study was to create various formulations of yogurt enriched with freeze-dried adjuncts, namely (i) probiotic *Lactobacillus plantarum* ATCC 14917 culture, and (ii) *L. plantarum* ATCC 14917 fermented black chokeberry juice, along with a commercial starter culture. The goal was to enhance functionality and optimize the nutritional value of the products. These new yogurt-style formulations were subsequently compared with commercially produced yogurt. All products demonstrated favorable physicochemical properties, and the probiotic strain consistently maintained viable levels exceeding 7 log cfu/g throughout the entire storage period. The fermented milk produced with the adjunct-free *L. plantarum* cells, as well as the yogurt produced with the proposed lactobacilli-fermented chokeberry juice, exhibited the highest lactic acid production (1.44 g/100 g yogurt by the end of storage). Levels of syneresis were observed at lower values in yogurt produced with freeze-dried fermented chokeberry juice. Yogurts prepared with the lactobacilli-fermented freeze-dried chokeberry juice displayed elevated total phenolic content and antioxidant capacity (25.74 µg GAE/g and 69.05 µmol TE/100 g, respectively). Furthermore, sensory tests revealed a distinctive fruity flavor in samples incorporating fermented juice. The results demonstrate that probiotic *L. plantarum*-fermented chokeberry juice enhances both the antioxidant capacity and the viability of beneficial bacteria in yogurt while it can be readily applied and commercialized, especially in the form of a freeze-dried formulation.

## 1. Introduction

Nowadays, promoting health and healthy habits are of major concern to consumers and functional foods are gaining increased recognition, popularity, and acceptance [1,2]. Specifically, fermented foods and their advantageous microbiota are anticipated to drive the growth of the functional food industry in the coming years [3]. Among the diverse range of fermented food products, probiotic foods emerge as a prominent category within the functional food industry [4,5,6]. Probiotics consist of living microorganisms that, when consumed in sufficient quantities (>10^6^ cfu/mL), offer health advantages to the host [4]. Even though the potential health effects of probiotics depend on a multitude of parameters, a key characteristic is to retain a sufficient number of live cells during consumption. Moreover, it is considered crucial that probiotic strains survive through the gastrointestinal tract and colonize the intestine to confer their beneficial health effects [4,5]. Within another perspective, probiotic cells inoculated for food fermentation can produce a wide range of bacterial metabolites depending on the food substrate [3,7]. Specifically, probiotic-fermented food can be a significant source of dietary postbiotics; a new term defined by the International Scientific Association for Probiotics and Prebiotics (ISAPP) as a preparation of inanimate [dead] microorganisms and/or their components that confers a health benefit on the host [8].

Fermentation is a bioprocess that involves controlled microbial activity and growth, commonly used for preserving foods such as milk. Yogurt is a commonly fermented dairy product, rich in various nutrients and minerals, while its consumption has increased globally due to its nutritional value and ease of digestion [9]. Specifically, yogurt has an increasing compound annual growth rate (6.9%), while its global market value is expected to reach USD 51 billion by year 2024 [10]. Therefore, innovation comprises one of the most significant challenges of the dairy industry to address adapting to consumer demands, while maintaining sustainable development. Likewise, fermented beverages are widely manufactured in the modern food industry as they provide specific organoleptic properties and unique bioactive compounds conferring health benefits [11,12,13,14,15,16].

Starter cultures play a crucial role in the production of fermented foods and beverages, contributing to nutritional value, functionality, flavor, and preservation. The advancements in modern microbiology have allowed the identification and application of specific starter cultures to achieve desirable attributes in food products [17]. The main application form of starter cultures in food fermentation is the lyophilized (freeze-dried) form, allowing for direct incorporation into food formulations [18]. This direct-to-set dried culture form is highly popular from an industrial perspective, as it eliminates in-plant subculturing, reduces the cost of bulk culture preparation, and protects against bacteriophage infection [4]. For example, the probiotic strain *Lactobacillus plantarum* has been recognized as a strain capable of enhancing the beneficial value of foods by providing the enhanced levels of bioactive compounds [19,20]. This capability positively influences the nutritional, sensory, and shelf-life attributes of fermented food products [4,11].

Yogurt is traditionally produced by milk fermentation with the bacterial strains *Lactobacillus delbrueckii* subsp. *bulgaricus* and *Streptococcus thermophilus* strains as the starter cultures, and represents one of the major dairy products for probiotic transfer [9]. Generally, yogurt can be fortified with a variety of bioactive compounds, offering a wide array of possibilities to support and enhance health, including strengthening the immune system, alleviating allergy symptoms, delivering antioxidant properties, averting cardiovascular conditions, increasing energy levels, and promoting cognitive functions [1,21]. Natural bioactive additives, such as fresh and dried fruits and fruit juices, are well known for their high nutritional value and bioactive content. They provide essential components for human nutrition, and their consumption is associated with numerous health benefits [11]. A recent trend involves combining probiotics and natural bioactive additives in yogurts to enhance their safety and nutritional value [22]. Examples of specific components added to yogurt for improved bioactive and functional properties include natural plant extracts like eugenol and cinnamaldehyde nanoemulsions [23], beta-carotene/soy protein isolate-containing lipid microparticles [24], green tea [25], bioactive peptides, gamma-aminobutyric acid [26], and fruits such as sea buckthorn berries [22] and black mulberries [27].

The beneficial synergies between fruit juices and bioactive substances, including probiotic bacteria and postbiotics, and their incorporation into dairy products, have the potential to initiate a new era of advancements in functional foods. For instance, probiotic cells added either as starter or adjunct cultures for food fermentation have been reported to produce phenolic compounds and short-chain fatty acids enhancing a product’s nutritional value [28]. Also, the effect of fruit and fruit juices on the growth of probiotics has been reported to be species as well as strain specific [11,12,15,22]. For instance, the fermentation of pomegranate juice by immobilized *Lactobacillus paracasei* on wheat bran resulted in the production of good-quality, potentially synbiotic beverages. This process led to an increase in total phenolic content (TPC) and the aromatic volatile profile [12]. Additionally, probiotic strains of *L. plantarum* and *L. acidophilus* demonstrated the ability to survive and utilize fruit juices as substrates for their cell synthesis, showing promise for probiotic-fortified fruit juice production [13]. In another study, the fermentation of strawberry juice by *L. plantarum* and *L. acidophilus* led to improvements in color, phenolics, and antioxidant properties in the resulting beverage [14]. Furthermore, the fermentation of jujube-wolfberry juice by *L. plantarum* enhanced its antioxidant activity [16]. Lastly, a novel potentially probiotic strain, *L. paracasei*, isolated from kefir grains, demonstrated high cell survival during fermentation and storage when evaluated for chokeberry juice fermentation. The fermented juice maintained its aroma complexity and exhibited increased TPC and antioxidant capacity compared to the non-fermented juice [15]. 

The utilization of freeze-dried fermented juices as carriers of functional components is a relatively recent concept, and there is ample room for further exploration into their impact on yogurt starter cultures and the nutritional value of yogurt. Recent studies have demonstrated improvements in the sensory properties and enhanced functionality of yogurts during fermentation and storage by incorporating freeze-dried mulberry juice [29], freeze-dried powders of maqui (*Aristotelia chilensis*) and murra (*Rubus ulmifolius*) ultrasound-assisted extracts [30], freeze-dried soybean and mung bean peel powders [31], among others. However, there are a lack of reported assessments regarding the physicochemical, antioxidant, microbial, and sensory attributes of yogurts enriched with probiotic-fermented *Aronia melanocarpa* (black chokeberry) juice. Black chokeberries, also known as aronia berries, are recognized for their diverse health benefits attributed to their high polyphenol levels and significant antioxidant capacity [15]. Additionally, it is noteworthy that Aronia berries are rich in anthocyanins, which exhibit health-promoting properties such as antioxidative, anti-inflammatory, and antibacterial activity, along with prebiotic effects [32].

Given the aforementioned research, the primary focus of this study was to evaluate the potential applications of the probiotic strain *L. plantarum* ATCC 14917, along with fermented chokeberry juice using the same strain, as freeze-dried adjuncts for the development of various yogurt formulations. The quality of the resulting yogurts was evaluated based on several parameters, including physicochemical properties (acidity, TPC, antioxidant activity), sensory attributes, as well as microbiological stability during storage.

## 2. Materials and Methods

### 2.1. Microbial Starter Cultures

The commercial ready-vat bacterial culture consisting of *S. thermophilus* and *L. delbrueckii* ssp. *bulgaricus* at a 2:1 proportion (Lyofast Y 436 A, Sacco Systems, Cadorago, Italy) was applied as starter culture for yogurt production [33]. The starter culture was activated by incubation in 10 mL of sterile skim milk at 43 °C for 1 h according to the instructions of the manufacturer. The probiotic strain *L. plantarum* ATCC 14917 obtained in lyophilized form (LGC Standards, Middlesex, UK), was activated and grown at 37 °C in de Man-Rogosa-Sharpe (MRS) liquid medium (Merck Darmstadt, Germany) in agitated (180 ± 5 rpm, of atmosphere approx. 5% CO_2_) conical flasks for 24 h according to the manufacturer instructions. All media were sterilized prior to use by autoclaving at 121 °C for 15 min (1–1.5 atm). The obtained wet cell mass was harvested by centrifugation (5000 rpm for 10 min, Sigma 3K12 centrifugation system, Bioblock Scientific, Illkirch Cedex, France), and subsequently frozen to −44 °C at a cooling rate of 5 °C/min for minimum cell viability loss [12]. Then, the harvested cell mass was freeze-dried for 48 h without cryoprotection (FreeZone 4.5 Freeze-Drying System, Labconco, Kansas City, MO, USA).

### 2.2. Black Chokeberry Juice Production and Fermentation

Black chokeberries (*Aronia melanocarpa*) were sourced fresh from a local organic farming producer in Nea Orestiada, located in the northeastern part of Evros, Thrace, Greece.

The black chokeberries were carefully selected and thoroughly washed with a sterile 1/4 ringer solution. Subsequently, the berries were crushed and homogenized by blending for 10 min in a common household blender [34]. The pulp was removed by a sterile cloth strainer and centrifuged (3000 rpm, 10 min). The primary chemical composition of *Aronia melanocarpa* juice was as follows, as determined in prior research: malic acid 4.71 g/L ± 0.05, sorbitol 20.0 g/L ± 0.2, glucose 11.05 g/L ± 0.2 and fructose 9.13 g/L ± 0.3 [15]. After extracting the juice, sterilized deionized water was added to adjust the initial sugar concentration (approximately 40 g/L), and the juice was then pasteurized (80 °C, 10 min) [35]. After cooling the black chokeberry juice to room temperature, 1 g (dry weight) of freeze-dried *L. plantarum* was suspended per 100 mL of juice. The viability of the *L. plantarum* strain was assessed to be 8.3 log cfu/mL through selective media counting [36] at the onset of fermentation. Subsequently, the juice underwent fermentation at 30 °C for 48 h, with simultaneous pH maintenance at 4.0 ± 0.2 achieved by adding Na_2_CO_3_ solution at various time intervals. Subsequently, each fermented juice was subjected to freeze-drying [12].

### 2.3. Novel Yogurt-Style Production

Yogurt-style products were prepared from pasteurized homogenized cow’s milk of Greek origin (pH 6.8 ± 0.1, 3.7% fat, 13.0% total solids) as a fermentation media. The milk was initially heated at 90 °C for 5 min, cooled at 40 °C, and subsequently divided into 5 equal portions of 100 mL each in sterile glass containers [33]. Five different formulations were prepared, namely: CY (commercial yogurt used as control sample) inoculated with yogurt starter culture (5% inoculum, *S. thermophilus* and *L. bulgaricus*); LPCY inoculated with yogurt starter culture and *L. plantarum* (5% inoculum, 1:1); LPY inoculated only with *L. plantarum* (5% inoculum); PDCY inoculated with yogurt starter culture and chokeberry juice fermented with *L. plantarum* (5% inoculum); and PDY inoculated with chokeberry juice fermented with *L. plantarum* (5% inoculum). The samples were incubated (40 ± 1 °C for approx. 5–5.5 h) and the acidification was monitored (Consort D130 system, Turnhout, Belgium) until a pH drop at 4.6 ± 0.1. When a pH drop was achieved, each fermented sample was placed for cold storage at 4 °C for 28 days.

### 2.4. Physicochemical Analysis 

Compositional analysis and pH were performed for each of the fermented milk samples after the 1st day of cold storage. Specifically, the samples were analyzed for total solids (method 990.19), ash (method 945.46), and fat (method 989.05) and using the methods of AOAC (2005). Fat and protein contents were determined by the Soxhlet and Kjeldahl methods, respectively. The pH values of samples were determined by direct immersion of the electrode using a digital pH meter (Hanna HI99161). 

High-performance liquid chromatography (HPLC) was used for sugar and organic acid quantification. In brief, lactose was determined on an HPLC system (Shimadzu, Kyoto, Japan) with an SCR-101N stainless steel column, an LC-9A pump, a CTO-10A oven (60 °C), and an RID-6A index detector. Lactic acid was determined on an HPLC system (Shimadzu, Kyoto, Japan) with a Shim-pack IC-A1 stainless-steel column, a LC-10A pump, a CTO-10A oven (40 °C) and a CDD-6A detector. For quantitative analysis, standard solutions of sugars and acids (Saint Louis, Misouri, USA, Sigma-Aldrich Ltd.) were prepared in ultrapure water (Milli-Q, Darmstadt, Germany, Merk) at various concentrations [33].

Syneresis (S%) was assessed on the first day of production to analyze the potential effects of supplements on the yogurt-style products during refrigerated storage. Samples weighing 10 g were placed in 15 mL Falcon tubes, and were then centrifuged (350× *g*, 20 min, 4 °C) and the separated serum was weighed. The level of syneresis was calculated using the following equation:

$$S\%=\frac{weight\;volume\;of\;supernatant}{Weight\;of\;fermented\;yogurt-style\;sample}\times 100$$

### 2.5. Microbiological Assessment and Viable Probiotic Cell Count

Microbiological analysis was conducted at various time intervals during the 28 days of cold storage (4 °C) according to the literature with small modifications [22]. Specifically, 10 g of each yogurt-style product was thoroughly homogenized in 90 mL of sterile Ringer solution (LABM, Heywood, Bury, UK) and serially diluted in sterile 0.1% (wt/vol) peptone water (Oxoid Ltd, Hampshire, UK). The microbial loads of each sample were determined by plating on selective media. Specifically, *S. thermophilus* was plated on M17 agar containing 1% lactose and incubated aerobically (40 °C, 72 h); *L. bulgaricus* was plated on de Man, Rogosa, Sharpe (MRS) agar with 10% sorbitol and incubated anaerobically (37 °C, 48 h); yeasts and molds were plated on Potato Dextrose Agar and incubated aerobically (30 °C for 72 h); coliforms were plated on Violet Red Bile agar after and incubated anaerobically (30 °C, 24 h); staphylococci were plated on Baird Parker agar after and incubated aerobically (37 °C, 24 h), and the petri dishes remained for another 24 h to assess the viability of *S. aureus* strains (black or grey colonies) [37] according to the manufacturer instructions (LABM, UK). 

Viable bacterial counts of *L. plantarum* were determined by plating on MRS agar with 10 mg/L of vancomycin antibiotic that promotes its growth against *L. bulgaricus* [38] and incubating aerobically (37 °C, 48 h).

All the above media were sterilized (121 °C, 15 min) before use. Cell counts were expressed as log cfu/g [33].

### 2.6. Antioxidant Capacity

Free radical-scavenging activity was determined using the free radical DPPH (2,2 diphenyl-1-picrylhydrazyl) method [39]. The DPPH radical scavenging activity was determined according to the following equation (Ac: the absorbance of the control solution, As: the absorbance of the test solution):

$$DPPH\;scavenging\;activity\%=\frac{Ac-As}{Ac}\times 100$$

Total phenolic content (TPC) was determined by the Folin–Ciocalteu method assessed accordingly to the literature [40]. Concentrations are expressed in Gallic Acid Equivalents (GAE).

### 2.7. Sensory Attributes

All yogurt-style samples (LPCY, LPY, PDCY, PDY) underwent sensory evaluations conducted by 10 adults (laboratory members; priorly trained) familiar with the consumption of dairy products and were compared to yogurt made using the commercial starter culture [33]. Approximately 50 g of samples produced the day before the evaluation were provided to the assessors. The assessors, both males and females aged 25 to 45 years who were non-smokers, were involved in the evaluation process. The samples were served in 50 mL transparent plastic cups, each numbered randomly with 3-digit codes. The sensory evaluation session took place in individual booths. They were provided with bread and low-mineral content water to cleanse their palate after each tasting. The panel was instructed to assign scores on a 0–10 scale (0 = unacceptable, 10 = exceptional) for attributes falling under the following main categories: aroma, fruit flavor, intensity of white color, texture, firm body, sour taste, overall flavor, and overall acceptability. At the conclusion of the rating scale, assessors were invited to provide any additional comments. 

### 2.8. Statistical Analysis

All fermentation experiments were carried out in triplicate. The results were analyzed using one-way analysis of variance (ANOVA). The different treatments were compared at the same storage period, and samples from the same treatment were compared during the same time. Duncan’s multiple range tests were applied in order to determine significant differences (coefficients, ANOVA tables, and significance); *p* value < 0.05 was considered statistically significant for all analyses. 

## 3. Results and Discussion

### 3.1. Impact of Incorporated Enriched Materials on the Physicochemical Characteristics of the Products

The results pertaining to the pH, lactic acid, and lactose content of the fermented milk samples during cold storage (4 °C) for 28 days are detailed in Table 1. Overall, a decrease in pH values was noted across all samples during storage. The reduction in pH during lactic acid milk fermentation is attributed to the breakdown of lactose into lactic acid, where a higher production of lactic acid corresponds to a lower pH value [33,41]. The pH values of samples containing the probiotic strain were detected at significantly lower levels compared to the control sample (CY). Furthermore, the incorporation of the probiotic strain in all respective fermented milk samples (LPCY, LPY, PDCY, and PDY) increased lactic acid production, resulting in lower pH values after fermentation and during cold storage, compared to the control (CY). This result is consistent with previous studies indicating that yogurt production with adjunct probiotic cultures shows heightened lactic acid production and lower pH values compared to commercial yogurt, attributable to the enhanced accumulation of the probiotic strain [33,42,43]. Milk fermentation by lactic acid bacteria involves the conversion of milk lactose, mainly to lactic acid (0.6–1%), along with other metabolites. Thus, a decline in pH during the initial periods of storage can be expected as a result of post-acidification. The most significant decrease in lactose, accompanied by a decline in pH, was noted in PDCY and PDY samples. Consequently, the trend toward lower pH is a result of increased lactic acid production and potentially other organic compounds, such as formic acid, acetaldehyde, and acetic acid [33,43,44].

The decrease in pH in all samples during storage, most likely due to the production of organic acids, is a result of the activity of lactic acid bacteria (LAB), which seem to remain active even at low temperatures. Notably, the samples containing the adjunct probiotic culture exhibited lower pH values, while the samples with the dried supplements displayed even lower pH levels and the highest lactic acid production. Specifically, by the end of storage period, the sample PDY recorded the lowest pH (4.24) followed by the PDCY sample (pH 4.27) with the other samples having a pH in the range of 4.48–4.35. The distinct acidification observed in fermented milk samples produced with the addition of fermented chokeberry juice with *L. plantarum* may be attributed to the stimulating effect of the phenolic compounds found in chokeberries on the metabolic activity of LAB. However, it should be noted that the pH of these samples remained within acceptable values. This result aligns with recent studies that have confirmed the positive influence of phenolic compounds on lactic acid fermentation [14,45]. 

The protein, fat, total solids, and ash content from the first day of production are presented in Table 2 and ranged between 3.3–3.6 (%), 3.5–3.6 (%), 16.6–16.9 (%), and 0.5–0.7 (%), respectively. The slight reduction in fat content in all samples could be ascribed to the lipolytic activity of microorganisms [46,47]. The protein content exhibited variation among the samples. Specifically, the control sample (CY) displayed a protein content similar to that of LPCY and LDY (3.5%), while the PDY sample had a slightly lower protein content (3.3%). In contrast, the PDCY sample had the lowest protein content among the samples, measuring at 3.0%.

Serum separation in yogurt and yogurt-style products is a critical factor that impacts the appearance and physical characteristics [48]. Yogurt as well as sour milk syneresis denotes the separation of whey on the surface of the product, which can happen either upon opening a yogurt container or in a sealed container. This separation is primarily attributed to (i) the higher concentration of whey protein compared to casein, and (ii) the lower concentrations of total solids, as well as (iii) changes in organic acids produced by viable LAB during storage [49]. According to the results of the current study, syneresis was influenced by the initial starter culture and acidification method, as well as the added powdered supplements (Table 2). The addition of chokeberry juice fermented with *L. plantarum* (PDCY, PDY) can enhance the total solid content providing higher consistency in fermented milk compared to commercial yogurt samples. Furthermore, as indicated by previous studies, the addition of fruits or supplements with high antioxidant activity has the potential to reduce serum separation and enhance the storage stability of fermented milk products [27,50].

### 3.2. Antioxidant Activity and Phenolic Content

Lactic acid fermentation is known to significantly enhance the antioxidant capacity, phenolic content, and flavor of fresh juice when lactic acid bacteria are applied for fermentation, as indicated by recent studies [11,51,52]. Likewise, the results of this study indicate that the addition of powdered supplements combined either with commercial starter culture or fermented milk (PDY, PDCY) boosted the levels of the antioxidant activity (approximately 69 µmol TE/100 g). In general, all samples fermented with the adjunct probiotic *L. paracasei* strain showed higher antioxidant capacity in contrast to plain yogurt, which reached a DPPH radical scavenging activity of 49 µmol TE/100 g. The results indicated that the probiotic strain increased the antioxidant capacity of the fermented milk products, while chokeberry components can also significantly enhance the antioxidant content either by the direct contribution of antioxidant compounds or thought bioconversion derived from LAB activity. 

The TPC appeared also enhanced in the samples produced with the chokeberry juice fermented with *L. plantarum* (PDY, PDCY) compared to all other samples. According to the literature, in terms of the contribution sources of bioactive polyphenols (phenolic acids, flavonols, anthocyanins, proanthocyanidins) included in chokeberry, the total antioxidant activity mainly depends on the contribution of free polyphenols [53,54]. As a result, it is evident that incorporating chokeberry as a supplement can contribute to the development of functional food by providing a natural source of polyphenols.

### 3.3. Microbial Stability of Yogurts-Style Products during Cold Storage

Microbial stability is crucial in preserving the physicochemical and organoleptic characteristics, as well as the safety of foods during storage, especially dairy products [55]. Microbial counts (log cfu/g) of possible spoilage microorganisms in the fermented milk samples were monitored throughout cold storage for 28 days. As noted, no spoilage or possible pathogenic microorganisms such as staphylococci, coliforms, enterobacteria, yeasts, or molds were detected during cold storage for 28 days in samples with the adjunct probiotic strain (Table 3). On the contrary, yeasts and molds were detected in the commercial yogurt samples after the 21st day of storage. This result is most likely due to the slightly higher pH values of the commercial yogurt, as well as the antagonistic effect of *L. plantarum* observed in all other samples (LPCY, LPY, PDCY, PDY). 

**Figure 1:**
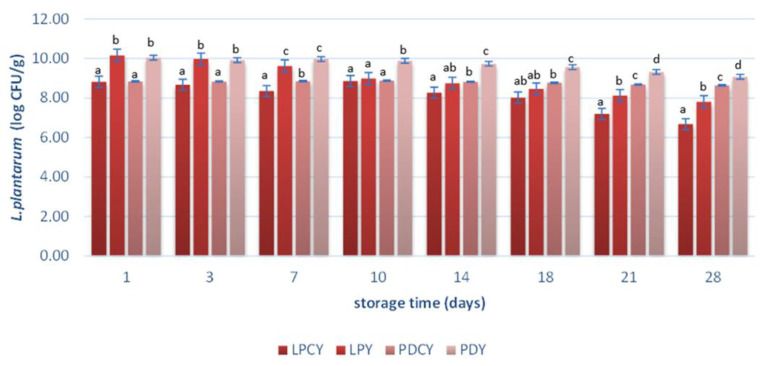
Viable cell counts (log cfu/g) of *L. plantarum*. LPCY: yogurt with commercial starter culture and *L. plantarum*; LPY: milk fermented with *L. plantarum*; PDCY: fermented milk with commercial yogurt starter culture and chokeberry juice fermented with *L. plantarum*; PDY: milk fermented with fermented chokeberry juice. Different superscript letters in the bars indicate statistically significant differences at the same time of analysis for the comparison each starter culture (MF-ANOVA with Tukey’s HSD multiple range test).

Microbial counts of *S. thermophilus*, *L. bulgaricus* (Table 3), and *L. plantarum* (Figure 1) were also monitored. The counts of lactic acid bacteria (LAB) during cold storage are intricately linked to nutritional and environmental factors [4]. As shown in Table 3, the incorporation of chokeberry juice fermented with *L. plantarum* resulted in an elevation of LAB counts in the PDCY sample during cold storage, contrasting with the commercial yogurt sample. More specifically, this resulted in higher counts of *S. thermophilus* and *L. bulgaricus*, exceeding 8 log cfu/mL on the 28th day of storage, whereas the control yogurt exhibited significantly lower viability at 7 log cfu/mL. This outcome can be attributed to the enrichment of nutrients and prebiotic oligosaccharides provided by the adjunct fermented chokeberry juice [22,56,57]. This finding is consistent with previous studies suggesting that prebiotic ingredients can enhance LAB viability in dairy products and gut microenvironment [4,33,44,58]. Moreover, the *S. thermophilus* and *L. bulgaricus* viable cell counts were found at a significantly lower concentration in sample LPCY, compared to the commercially produced yogurt samples (Table 3). This could be attributed to the antagonistic effect of the probiotic *L. plantarum* culture against other bacteria during milk fermentation [33,41]. In addition, the effect of low temperature on *S. thermophilus* and *L. bulgaricus* can affect their viability as they belong to thermophilic bacteria (optimum temperature 40–45 °C).

It has been reported that phenolic compounds at relatively high concentrations can inhibit the growth of bacteria [59]. In this study, higher viable counts of both *S. thermophilus* and *L. bulgaricus* were found in the PDCY sample from the first till the last days of storage, although these samples were enriched in phenolic compounds from the fermented chokeberry juice. On the other hand, samples produced with the commercial starter culture (CY and LPCY) showed significantly lower viability after the first days of production compared to PDCY samples. This may be attributed to the high phenolic content of PDCY samples, which, in line with previous studies, can promote the growth of *S. thermophilus* and *Lactobacillus* spp. during milk fermentation [25]. 

### 3.4. Viability of L. plantarum and Possible Beneficial Effects

Figure 1 illustrates the population of the probiotic strain *L. plantarum* in samples LPCY, LPY, PDCY, and PDY during cold storage (4 °C) at various time intervals (days 1, 3, 7, 10, 14, 18, 21, 28). Well-documented, unique health benefits may arise from novel and unconventional sources of probiotics, which can produce bioactive compounds contributing to well-being, and therefore, it is crucial to maintain the strains viability throughout storage [4,60]. It is essential to note that the initial viability of *L. plantarum* was recorded at 8.8 log cfu/mL after juice fermentation (48 h), with a slight decrease in viability (8.6 log cfu/mL) observed after freeze-drying of the fermented juice. This result is possibly attributed to the harsh conditions of freeze-drying to which the fermented juice was subjected without the use of cryoprotectants. Regarding the viability of the probiotic strain in fermented milk samples, it is important to note that the *L. plantarum* strain consistently maintained levels above 8 log cfu/g throughout the storage period in all samples.

All yogurt-style products meet the criteria to be considered probiotic, aligning with the recommended levels of viable probiotic cell counts at the time of consumption [4]. This result is noteworthy, as prior studies suggest that the *L. plantarum* strain is not as well suited for milk fermentation as other probiotic strains since genomic and milk fermentation test verified low-level lactic acid production in this particular strain [19]. Conversely, it was demonstrated to be highly suitable for chokeberry juice fermentation, and its adjunct addition in milk alongside chokeberry was proven to be highly successful. In relation to the above observation, a significant decline (*p* < 0.05) in probiotic cell counts was observed at the end of the storage period in samples produced with *L. plantarum* as the starter culture (LPY) and in samples produced with both *L. plantarum* and the commercial yogurt culture (LPCY), compared to samples produced with chokeberry juice fermented with *L. plantarum* (PDCY). This decrease in cell counts can be attributed to the acidic environment of fermented milk samples, which adversely affects the viability of free bacterial cells, leading to their reduction [33]. Furthermore, this decrease may be attributed to the deficiency of LacZ/LacLM genes, as confirmed by previous studies [19]. This deficiency can result in the incapacity of *L. plantarum* to ferment lactose, especially during cold storage, thus leading to a loss of viability. In contrast, samples produced with chokeberry juice and *L. plantarum* (PDCY), as well as milk fermented with chokeberry juice and *L. plantarum* (PDY), demonstrated remarkable stability in viable probiotic cell counts throughout the entire storage period (4 °C, 28 days). This result may be attributed to the increased phenolic content of samples produced with the powdered supplements, as anthocyanins provided by chokeberry may promote the growth of lactic acid bacteria (LAB) due to their prebiotic activities [57,61]. 

In the recent literature, it is also suggested that prebiotics may contribute to the protection of probiotic cells against acidic and harsh environmental conditions during the production and storage of dairy products [5]. Likewise, in this study, the viability of *L. plantarum* could be enhanced by prebiotic ingredients present in the dried chokeberry juice providing a synbiotic supplement [62]. Importantly, prior studies have proposed a synergistic interaction between phenolic compounds and probiotic bacteria, resulting in an improved antioxidant capacity and the colonization of probiotic cells within the gastrointestinal tract [63]. These synergistic interactions may also be associated with postbiotic effects, which involve the production of small metabolites by bacteria during their life cycles. These metabolites play a crucial role in regulating bacterial growth and cell communication. Additionally, they contribute to the growth of beneficial bacteria and offer protection against various stresses [64]. 

Building on these findings, the present study suggests a blend of probiotic bacteria and chokeberry juice, known for its phenolic compounds, as a powdered supplement for yogurt-style products’ manufacturing. This strategy, incorporating the components of chokeberry juice, is envisioned to contribute to the sustained high survival rates of probiotics in the resultant dairy products. Additionally, previous research has highlighted the diverse functional properties of *L. plantarum* strains in the food industry. These properties include enhancing nutritional quality, flavor characteristics, antioxidant and antimicrobial activities, and extending the shelf life of foods, while also reducing undesirable compounds [65]. However, it is important to highlight that for populations with allergies or intolerances to dairy-based products, the fermentation of fruit juices with Lactobacillus isolates may serve as an alternative. Probiotic-fortified fruit juices, such as chokeberry juice, can be explored as functional and healthy beverages delivering probiotics, as demonstrated in previous studies [13,15].

### 3.5. Sensory Evaluation

**Figure 2:**
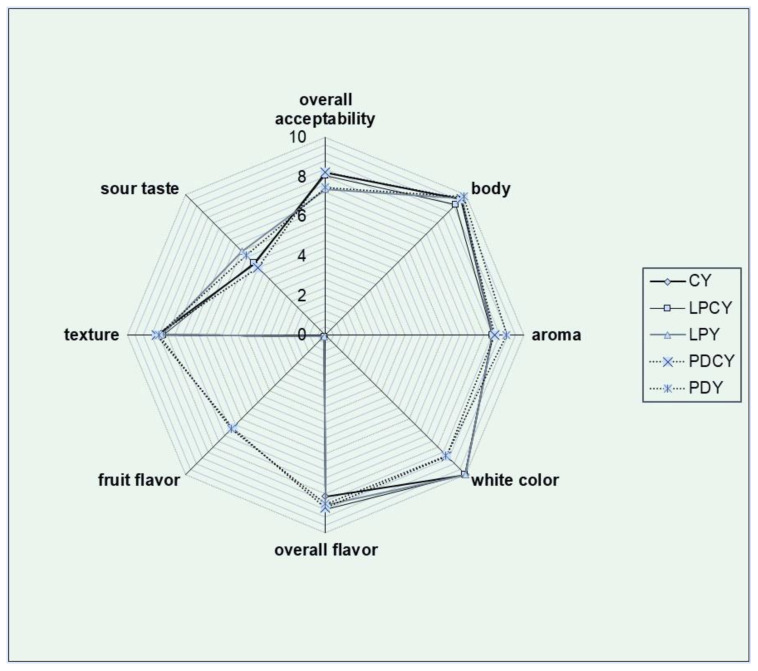
Radar plot for the sensory attributes of samples. CY: yogurt with commercial starter culture. LPCY: milk fermented with commercial yogurt culture and *L. plantarum*; LPY: milk fermented with *L. plantarum*; PDCY: milk fermented with commercial yogurt culture and chokeberry juice fermented with *L. plantarum*; PDY: milk fermented with fermented chokeberry juice.

Overall, all samples garnered high acceptance scores and were marked by a favorable total impression, with no off-flavors detected (Figure 2). The samples prepared with fermented chokeberry juice containing *L. plantarum* stood out for its exceptional aroma and fruit flavor. This characteristic could be attributed to the abundance of phenolic compounds, recognized as significant constituents in plant-origin food products. Phenolic compounds are related to the significant sensory characteristics of foods such as flavor, astringency, and color [66]. According to the results presented in Figure 2, the color of fermented milk samples was significantly affected by the addition of the fermented chokeberry juice in PDY and PDCY samples. The abundance of phenolic compounds (Table 2) in samples PDCY (Figure 2) and PDY (Figure 2), provided by the initial juice content, can contribute to the flavor of fermented milk highlighting its unique aromatic characteristics. Similarly, volatile phenols generated through the metabolic processes of probiotic or yogurt cultures can contribute to an enriched flavor and aromatic profile, aligning with findings from prior studies [66].

The texture of all samples received high evaluation scores, likely because the syneresis values remained within acceptable ranges. This could be attributed to the presence of LAB metabolites, which may enhance the firmness of the product’s texture. For example, probiotic and LAB strains are recognized for their exopolysaccharide production, which can contribute to the texture of fermented milk, reducing syneresis and improving the overall mouthfeel and body of the product [67]. Similarly, it has been indicated that yogurt texture can be enhanced through the production of exopolysaccharides, which interact with the water in the products, creating a gel-like structure Guzel-Seydim, Sezgin, and Seydim [67]. The elevated texture scores observed in the samples of this study may also be attributed to other constituents, such as total solids. Notably, the slightly higher texture scores of PDCY and PDY (yogurts with fermented chokeberry juice with *L. plantarum*) compared to other samples could be associated with carbohydrates and fibers derived from the fermented juice powder. These components could play a significant role in improving yogurt texture. Quality characteristics like these are crucial for the food industry, as they can have substantial effects on the viscosity and firmness of fermented milk products. More crucially, in today’s context, consumers are increasingly receptive to trying novel flavor combinations, particularly those integrating ingredients that can provide beneficial health effects. This shift is emphasized by the increased significance placed on health, especially in the aftermath of the COVID-19 pandemic [68].

## 4. Conclusions

Freeze-dried fermented chokeberry juice with the probiotic strain *L. plantarum* ATCC 14917 was effectively integrated in the production of functional fermented milk products. The resulting innovative yogurt-style products maintained robust probiotic culture viabilities and an enhanced phenolic profile throughout a 4-week storage period. Notably, in all cases where *L. plantarum* was incorporated, the achieved viabilities consistently surpassed the threshold (10^6^–10^7^ cfu/mL) required to confer the health benefits of a specific probiotic food. Furthermore, the novel yogurt-style products containing the incorporated fermented chokeberry juice exhibited microbiological safety throughout the entire storage period. This novel supplement, exemplified by freeze-dried fermented chokeberry juice, not only meets the demand as a probiotic product but also merges bioactive phenolic components from the original juice with those generated by LAB metabolism. Readily deployable functional supplements are notably attractive to the food industry, especially in the post-pandemic era. Hence, the groundbreaking functional yogurt-style products developed in this study showcase substantial potential for commercialization in the dairy industry, offering a blend of advantageous antioxidant, prebiotic, probiotic, and postbiotic attributes. Finally, future research endeavors should focus on delving deeper into the nutritional aspects and interactions posed by the expression of LAB genes in novel products. This is essential to fully understand the metabolic compounds occurring in the final products, as well as to uncover the potential health effects and benefits associated with the consumption of these products.

## Figures and Tables

**Table 1:** Means and standard deviations of pH, lactose, and lactic acid in fermented milk samples during refrigerated storage at 4 °C for 28 days.

	Storage Time (Days)	CY	LPCY	LPY	PDCY	PDY
pH	1	4.61 ^a^ ± 0.08	4.59 ^a^ ± 0.09	4.58 ^a^ ± 0.12	4.57 ^a^ ± 0.05	4.56 ^a^ ± 0.05
	7	4.56 ^a^ ± 0.10	4.51 ^a^ ± 0.17	4.49 ^a^ ± 0.10	4.48 ^a^ ± 0.10	4.39 ^a^ ± 0.08
	14	4.53 ^b^ ± 0.05	4.45 ^b^ ± 0.05	4.44 ^ab^ ± 0.11	4.42 ^ab^ ± 0.07	4.31 ^a^ ± 0.10
	21	4.51 ^b^ ± 0.11	4.42 ^b^ ± 0.08	4.39 ^ab^ ± 0.10	4.33 ^ab^ ± 0.05	4.26 ^a^ ± 0.05
	28	4.48 ^c^ ± 0.03	4.38 ^bc^ ± 0.05	4.35 ^ab^ ± 0.08	4.27 ^ab^ ± 0.09	4.24 ^a^ ± 0.05
Lactose (g/100 g of product)	1	2.27 ^a^ ± 0.08	2.25 ^a^ ± 0.09	2.31 ^a^ ± 0.09	2.45 ^a^ ± 0.11	2.43 ^a^ ± 0.11
	7	1.93 ^a^ ± 0.11	1.99 ^a^ ± 0.07	2.05 ^a^ ± 0.11	2.03 ^a^ ± 0.10	2.12 ^a^ ± 0.10
	15	1.90 ^a^ ± 0.13	1.38 ^bc^ ± 0.11	1.69 ^b^ ± 0.08	1.47 ^bc^ ± 0.08	1.77 ^a^ ± 0.14
	21	1.84 ^b^ ± 0.09	1.18 ^a^ ± 0.07	1.30 ^a^ ± 0.08	1.11 ^a^ ± 0.12	1.19 ^a^ ± 0.13
	28	1.72 ^b^ ± 0.05	0.99 ^a^ ± 0.05	1.12 ^a^ ± 0.07	0.95 ^a^ ± 0.09	1.05 ^a^ ± 0.10
Lactic acid (g/100 g of product)	1	0.85 ^a^ ± 0.04	0.87 ^ab^ ± 0.11	0.91 ^a^ ± 0.05	0.88 ^a^ ± 0.05	0.99 ^ab^ ± 0.05
	7	1.12 ^ab^ ± 0.05	1.15 ^ab^ ± 0.05	1.04 ^ab^ ± 0.05	1.14 ^a^ ± 0.03	1.05 ^ab^ ± 0.03
	15	1.19 ^ab^ ± 0.05	1.22 ^ab^ ± 0.05	1.11 ^ab^ ± 0.06	1.25 ^ab^ ± 0.05	1.10 ^a^ ± 0.04
	21	1.23 ^b^ ± 0.04	1.34 ^b^ ± 0.06	1.18 ^ab^ ± 0.05	1.31 ^b^ ± 0.05	1.13 ^a^ ± 0.05
	28	1.31 ^b^ ± 0.05	1.44 ^b^ ± 0.05	1.24 ^a^ ± 0.03	1.44 ^b^ ± 0.03	1.17 ^a^ ± 0.04

The results are expressed as mean (*n* = 3) ± standard deviations; CY: yogurt with commercial starter culture; LPCY: fermented milk with commercial yogurt starter culture and *L. plantarum*; LPY: milk fermented with *L. plantarum*; PDCY: fermented milk with commercial yogurt starter culture and chokeberry juice fermented with *L. plantarum*; PDY: fermented milk with fermented chokeberry juice. Different superscript letters in rows indicate statistically significant differences at the same time of analysis for each starter culture (MF-ANOVA with Tukey’s HSD multiple range test).

**Table 2:** Physicochemical properties of fermented milk samples (first day of production).

Fermented Milk	Protein	Total Solids	Ash	Fat	Syneresis	TPC	DPPH
	(% wt)	(% wt)	(% wt)	(% wt)	%	(µg GAE/g)	(µmol TE/100 g)
CY	3.52 ^c^ ± 0.05	15.90 ^b^ ± 0.11	0.57 ^a^ ± 0.08	3.57 ^a^ ± 0.11	23.31 ^c^ ± 0.28	15.18 ^a^ ± 0.76	49.12 ^a^ ± 0.35
LPCY	3.54 ^c^ ± 0.07	15.89 ^b^ ± 0.15	0.61 ^a^ ± 0.05	3.69 ^a^ ± 0.07	24.13 ^d^ ± 0.14	16.20 ^a^ ± 0.51	61.40 ^b^ ± 0.19
LPY	3.48 ^c^ ± 0.05	15.77 ^b^ ± 0.11	0.52 ^a^ ± 0.03	3.65 ^a^ ± 0.05	24.19 ^d^ ± 0.10	15.24 ^a^ ± 0.87	51.72 ^a^ ± 0.31
PDCY	3.09 ^a^ ± 0.07	15.99 ^b^ ± 0.10	0.76 ^b^ ± 0.05	3.61 ^a^ ± 0.05	22.11 ^a^ ± 0.10	25.74 ^b^ ± 1.13	69.05 ^c^ ± 1.07
PDY	3.30 ^b^ ± 0.06	15.51 ^a^ ± 0.14	0.56 ^a^ ± 0.04	3.57 ^a^ ± 0.04	22.51 ^b^ ± 0.10	15.81 ^a^ ± 0.99	68.42 ^c^ ± 0.95

The results are expressed as mean (*n* = 3) ± standard deviations; TPC: total phenolic content; CY: yogurt with commercial starter culture; LPCY: yogurt with commercial starter culture and *L. plantarum*; LPY: milk fermented with *L. plantarum*; PDCY: Milk fermented with commercial yogurt starter culture and chokeberry juice fermented with *L. plantarum*; PDY: milk fermented with chokeberry juice fermented with *L. plantarum*. Different superscript letters in columns indicate statistically significant differences at the same parameter of analysis for each starter culture (MF-ANOVA with Tukey’s HSD multiple range test).

**Table 3:** Microbial counts of samples during cold storage (4 °C) for 28 days.

Fermented Milk	Storage Time	*S. thermophilus*	*L. bulgaricus*	Yeasts and Molds
	(Days)	(log cfu/g)		
CY	1	8.43 ^a^ ± 0.16	8.77 ^ab^ ± 0.12	nd
	7	8.14 ^a^ ± 0.25	8.44 ^b^ ± 0.23	nd
	14	7.66 ^b^ ± 0.11	8.19 ^b^ ± 0.11	nd
	21	7.21 ^b^ ± 0.10	7.74 ^b^ ± 0.14	nd
	28	7.11 ^b^ ± 0.10	7.26 ^b^ ± 0.11	1.06 ^a^ ± 0.10
LPCY	1	8.45 ^a^ ± 0.23	8.56 ^ab^ ± 0.11	nd
	7	8.07 ^a^ ± 0.19	7.17 ^a^ ± 0.09	nd
	14	7.12 ^a^ ± 0.14	6.64 ^a^ ± 0.28	nd
	21	6.42 ^a^ ± 0.11	6.57 ^a^ ± 0.10	nd
	28	6.06 ^a^ ± 0.08	6.34 ^a^ ± 0.12	nd
LPY	1	nd	nd	nd
	7	nd	nd	nd
	14	nd	nd	nd
	21	nd	nd	nd
	28	nd	nd	nd
PDCY	1	8.85 ^b^ ± 0.11	9.41 ^b^ ± 0.13	nd
	7	8.82 ^b^ ± 0.09	9.32 ^c^ ± 0.11	nd
	14	8.86 ^c^ ± 0.12	9.45 ^c^ ± 0.21	nd
	21	8.63 ^c^ ± 0.13	8.92 ^c^ ± 0.15	nd
	28	8.49 ^c^ ± 0.07	8.67 ^c^ ± 0.09	nd
PDY	1	nd	nd	nd
	7	nd	nd	nd
	14	nd	nd	nd
	21	nd	nd	nd
	28	nd	nd	1.10 ^a^ ± 0.14

The results are expressed as mean (*n* = 3) ± standard deviations; nd: not detected; CY: yogurt with commercial starter culture; LPCY: fermented milk with commercial yogurt starter culture and *L. plantarum*; LPY: milk fermented with *L. plantarum*; PDCY: fermented milk with commercial yogurt starter culture and chokeberry juice fermented with *L. plantarum*; PDY: fermented milk with chokeberry juice fermented with *L. plantarum*. Different superscript letters in columns indicate statistically significant differences at the same time of analysis for the comparison of each starter culture (MF-ANOVA with Tukey’s HSD multiple range test).

## Data Availability

The data are contained within the article.
